# Antibody Avidity Profiles as Diagnostic Biomarkers in Differentiating Acute and Chronic *Anisakis simplex*—Related Allergic Diseases

**DOI:** 10.3390/antib15010013

**Published:** 2026-02-06

**Authors:** Juan González-Fernández, Laura Ullate, Marta Rodero, Alvaro Daschner, Carmen Cuéllar

**Affiliations:** 1 Departamento de Microbiología y Parasitología, Facultad de Farmacia, Universidad Complutense de Madrid, 28040 Madrid, Spain; ullate.laura@gmail.com (L.U.); mrodero@ucm.es (M.R.); 2 Instituto de Investigación Sanitaria (IIS)—Servicio de Alergia, Hospital Universitario de La Princesa, 28006 Madrid, Spain; alvarodaschner@gmail.com

**Keywords:** *Anisakis simplex*, immunoglobulin avidity, IgE, IgG4, chronic urticarial, gastroallergic anisakiasis, ELISA, Western blot, parasite allergy, antibody maturation

## Abstract

Background/Objectives: Allergic features of anisakiasis, caused by ingestion of third-stage larvae of *Anisakis simplex* via raw or undercooked fish, manifest clinically as acute gastroallergic anisakiasis (GAA) or chronic urticaria with *Anisakis* sensitization (CU+). Differentiating these clinical phenotypes remains challenging. This study aimed to evaluate the maturation and avidity of specific antibodies (IgE, IgG4, IgG, and IgA) as biomarkers for discriminating between acute and chronic forms of anisakiasis. Methods: A prospective cohort of 65 patients from Madrid, Spain, was classified into three groups: GAA (n = 22), CU+ (n = 22), and chronic urticaria without sensitization (CU−, n = 21). Serum samples were analyzed for antigen-specific immunoglobulins using ELISA and Western blot. Avidity indices (AIs) were quantified through urea dissociation assays. Statistical comparisons and correlation analyses were performed to associate antibody avidity with clinical phenotype and demographic variables. Results: GAA patients exhibited significantly lower IgE avidity indices compared to CU+ individuals (mean AI: 79.9% vs. 88.5%), indicating a less mature IgE response during acute infection. Conversely, IgG4 and IgG avidity were elevated in GAA relative to CU+, reflecting an active but transient immune response. IgA antibodies were detected in both groups, although avidity differences lacked discriminatory capacity. No sex- or age-related differences in antibody avidity were observed. Longitudinal follow-up of GAA patients demonstrated an increase in IgE avidity over time. Conclusions: Quantitative assessment of antibody avidity, particularly for IgE and IgG4, enhances understanding of *A. simplex* immunopathogenesis and serves as a valuable biomarker for distinguishing acute from chronic clinical presentations. These findings support the use of avidity indices in the diagnosis, staging, and clinical management of anisakiasis.

## 1. Introduction

*Anisakis simplex* (Rudolphi, 1809) infection, a fishborne zoonosis of growing concern, has gained increasing public health relevance in regions with high consumption of raw or undercooked seafood. Over recent decades, its incidence has risen in parallel with changing dietary habits and improved clinical recognition. Beyond its well-established gastrointestinal manifestations, *A. simplex* is now recognized as a major elicitor of IgE-mediated allergic disorders, encompassing a wide clinical spectrum that ranges from acute gastroallergic reactions to chronic urticaria and, in severe cases, anaphylaxis.

*A. simplex* infection elicits a highly heterogeneous immune response, associated with clinical phenotype and a patient’s history of exposure [1,2,3]. Acute gastroallergic anisakiasis (GAA) is the clinical form most commonly associated with immediate hypersensitivity manifestations, including urticaria, angioedema, or anaphylaxis, which occur shortly after the penetration of viable larvae into the gastric mucosa. This condition is characterized by the robust, polyclonal activation of the immune response, evidenced by marked increases in *Anisakis*-specific IgE, IgG, IgG4, IgA, and IgM antibody levels. In contrast, other forms of anisakiasis or chronic exposure to non-viable *Anisakis* antigens, often related to dietary habits, may lead to IgE sensitization in the absence of overt clinical symptoms, resulting in a more persistent but less intense immune response. This diagnostic complexity is further compounded by significant cross-reactivity with other invertebrate allergens, such as mites and crustaceans.

Beyond the classical IgE response, the synthesis of IgG, IgG4, and IgA in GAA patients reflects the broad, helminth-related immune profile seen in *Anisakis* infection [3]. Recent data have highlighted the diagnostic value of non-IgE antibodies, especially IgA and IgG4, showing that both their concentration and ratio enhance discrimination between acute, chronic, and sensitized phenotypes [4]. In this context, elevated IgA levels and increased IgA/IgG4 ratios observed in selected patient groups suggest that assessing both antibody concentration and binding strength may improve disease staging, as antibody avidity represents a functional correlate of immune maturation. Consequently, the interaction and avidity of these isotypes may play a key role in modulating the balance between allergy and tolerance in anisakiasis. Advanced immunoassays such as ELISA and Western blotting enable precise quantification of antibody avidity and the identification of immunodominant antigenic fractions. The process of affinity maturation, a central mechanism driving the development of high-affinity antibodies, plays a pivotal role in shaping host immunity during parasitic infections. Several recent studies [5,6,7,8] have highlighted this phenomenon, showing that the assessment of IgG avidity serves as a reliable indicator for staging chronic parasitic diseases. These findings also emphasize the potential of extending avidity measurements to other isotypes, including IgA and IgG4, to better capture the complexity of humoral immune adaptation. Kaneva et al. [7] demonstrated the diagnostic utility of non-IgE antibody avidity in distinguishing acute from chronic parasitic infections, while Tork et al. [8] confirmed that IgG avidity effectively differentiates stages of *Toxoplasma gondii* infection. Collectively, these studies reinforce the broad applicability of avidity-based analyses in parasitic disease diagnostics.

Building on this concept, our group pioneered the examination of immunoglobulin avidity profiles in GAA and CU+ patients (chronic urticaria with *Anisakis* sensitization), uncovering distinctive signatures that mirror the balance between allergic reactivity and immunological tolerance. Specifically, GAA patients exhibited higher IgG and lower IgE avidity, with an inverse correlation between IgE avidity and both specific IgE and IgG4 levels [9]. Moreover, IgE avidity correlated with dietary fish intake and the time elapsed since the last parasite exposure, highlighting the dynamic modulation of antibody quality by antigen persistence and immune regulation. These findings suggest that evaluating IgG4 and IgA avidity alongside IgE could markedly enhance the specificity of *A. simplex* serodiagnosis, distinguishing previous exposure from current infection or allergy. Ultimately, antibody avidity profiles hold promise as sensitive biomarkers reflecting not only disease stage and prognosis but also the immune system’s shifting equilibrium between hypersensitivity and tolerance.

The principal objective of this study is to examine the maturation of specific antibodies (IgG, IgG4, IgE, and IgA) in anisakiasis, with an emphasis on the diagnostic value of low IgA/IgG avidity as a marker of recent infection. Using ELISA and Western blot, sera from patients with GAA, CU+ patients, and control CU− subjects (chronic urticaria without *Anisakis* sensitization) were analyzed. This study additionally aims to identify specific immunodominant antigens responsible for high-avidity immunoglobulin responses in order to refine serological diagnosis and deepen understanding of the immunopathology associated with *A. simplex* allergy.

## 2. Materials and Methods

### 2.1. Serum Samples

The study population was prospectively enrolled during the same recruitment period from a single geographic area (Madrid, Spain), following the diagnostic criteria established by González-Fernández et al. [4]. Participants were assigned to three cohorts: gastro-allergic anisakiasis (GAA; n = 22), chronic urticaria with *Anisakis*-specific sensitization (CU+; n = 22), and chronic urticaria without *Anisakis* sensitization (CU−; n = 21). Patients with CU− were included as negative controls because they were diagnosed in the Allergy Service (Hospital Universitario de La Princesa) as having chronic spontaneous urticaria with negative *Anisakis*-specific IgE, as assessed by both ImmunoCAP and skin prick testing. Importantly, although these patients were seronegative for *Anisakis*-specific IgE, they could still harbor *Anisakis*-specific antibodies of other isotypes, which justified their inclusion for the evaluation of non-IgE antibody responses.

All subjects provided written informed consent prior to inclusion. The study protocol was approved by the Institutional Review Board of the University Hospital La Princesa, Madrid (Protocol No. PI-515-07/04/11). These serum samples had been previously employed in related investigations [4] (Figure S1; Table S1).

### 2.2. Determination of Specific Antibodies by ELISA

Flat-bottom 96-well ELISA plates (Costar, Corning Inc., Corning, NY, USA) were coated overnight at 4 °C with 100 µL per well of *A. simplex* total larval antigen at 10 µg/mL, as determined by the Bradford protein assay. After washing with PBS containing 0.05% Tween 20 (PBS-T), plates were blocked with PBS containing 0.1% BSA for 1 h at 37 °C. Serum samples diluted in PBS-T with BSA were added in duplicate (1:100 for IgG4, IgG, and IgA; 1:2 for IgE) and incubated for 2 h at 37 °C.

Following washes, HRP-conjugated anti-human antibodies were added: anti-IgG4 (1:1000; SouthernBiotech, Birmingham, AL, USA), anti-IgG (1:8000; Biosource, Camarillo, CA, USA), or anti-IgA (1:3000; Biosource, Camarillo, CA, USA), and incubated for 1 h at 37 °C. For IgE detection, wells were first incubated with a mouse anti-human IgE antibody (IgG1, clone E21A11; 1:1000; Ingenasa, Madrid, Spain), followed by HRP-conjugated goat anti-mouse IgG1 (1:1000; Invitrogen, Eugene, OR, USA).

The colorimetric reaction was developed using o-phenylenediamine at 0.04% in phosphate-citrate buffer (pH 5.0) with 0.04% H_2_O_2_, stopped with sulfuric acid, and read at 490 nm. Antigen-free controls were included to correct for non-specific binding [4].

For detection of antibodies to larval excretory-secretory (ES) antigens, plates were prepared as above but coated with *A. simplex* ES antigen (1 µg/mL). The subsequent ELISA steps followed the same procedure as for the total larval antigen assay [4].

### 2.3. Determination of Avidity Index (AI) by ELISA

ELISA plates were coated overnight at 4 °C with either *A. simplex* total larval or ES antigen. After washing, plates were incubated with 6 M urea in PBS for 30 min at room temperature. Following an additional wash and blocking with BSA, serum samples were added in quadruplicate. Two replicates were treated with 6 M urea for 30 min, while the other two were incubated in PBS as controls. Plates were then processed as described in Section 2.2. Determination of Specific Antibodies by ELISA section, including incubation with HRP-conjugated secondary antibodies, OPD/H_2_O_2_ substrate development and absorbance reading at 490 nm.

Avidity Index (AI) values were calculated as (OD_1_/OD_2_) × 100, where OD_1_ and OD_2_ represent the mean optical densities of urea-treated and untreated wells, respectively, after subtraction of background binding to BSA. AI values > 50% were interpreted as indicative of high-avidity antibodies [5,7,10,11]. This parameter distinguishes acute from chronic or past exposure stages [12].

### 2.4. Immunorecognition Patterns by Western Blot

A total of 400 µg of *A. simplex* total larval antigen per lane was electrophoresed on 12.5% SDS-PAGE gels together with molecular weight markers (10–250 kDa; Precision Plus Protein™ Kaleidoscope™ Standards, Bio-Rad, CA, USA). Proteins were transferred onto 0.22 µm nitrocellulose membranes (Bio-Rad, Hercules, CA, USA; catalog no. 162-0112) using a Mini Trans-Blot Electrophoretic Transfer Cell (Bio-Rad) at 100 V for 1 h in Tris-glycine-methanol buffer.

Membranes were blocked overnight at 4 °C with 5% skimmed milk in PBS, washed three times for 5 min each with PBS-T before being cut into strips. Each strip was incubated with 800 µL of serum diluted 1:25 in 1% skimmed milk, 0.05% PBS-T for 3 h at 25 °C. After washing, strips were incubated with HRP-conjugated anti-human IgG4 (1:500), IgA (1:3000), or IgG (1:8000) for 2 h at 25 °C. Bands were visualized using 0.006% H_2_O_2_ and 0.05% DAB in PBS for 10 min in the dark, and the reaction was stopped with distilled water.

### 2.5. Determination of Avidity Index by Western Blot

Following protein transfer, membranes were incubated with 6 M urea for 30 min at room temperature, rinsed three times in 0.05% PBS-T, and blocked overnight at 4 °C with 5% skimmed milk in PBS. After washing, membranes were cut into strips and incubated in duplicate with 800 µL of serum diluted 1:25 in 1% skimmed milk–PBS-T for 3 h at room temperature.

One strip of each pair was treated with 6 M urea for 30 min, while the control strip was incubated in PBS-T. After treatment, strips were washed and processed as described above for immunoblot detection.

### 2.6. Statistical Analysis

Statistical analyses were performed using GraphPad Prism version 6.0 for Windows. Data are expressed as mean ± standard deviation (SD). Normality of distributions was evaluated using the Kolmogorov–Smirnov test with a *p*-value threshold of 0.05. Comparisons among three groups were carried out using one-way ANOVA followed by Bonferroni’s post hoc test for normally distributed data, or the Kruskal–Wallis test for nonparametric data. Comparisons between two groups employed Student’s *t*-test or Mann–Whitney *U* test as appropriate.

Correlations were analyzed using Pearson’s (parametric) or Spearman’s (nonparametric) correlation coefficients. A *p*-value < 0.05 was considered statistically significant.

## 3. Results

### 3.1. Avidity of IgE Against Anisakis simplex Total Larval Antigen (ELISA)

#### 3.1.1. Cross-Sectional Analysis

The avidity index (AI) of specific IgE against the *A. simplex* total larval antigen was determined by ELISA in sera from three groups: gastro-allergic anisakiasis (GAA), chronic urticaria with *Anisakis* sensitization (CU+), and chronic urticaria without *Anisakis* sensitization (CU−). Only sera exhibiting optical density (OD) values above 0.077 were included in the analysis. This threshold corresponded to the mean OD obtained from 80 negative sera tested against the same antigen. CU− patients were included as negative controls, as previously stated, but were also analyzed in the *Anisakis*-specific IgE avidity study because the ELISA method used in this work differs from the ImmunoCAP system routinely employed for hospital diagnosis. The detection of high-avidity IgE antibodies in some CU− sera is compatible with the presence of low-titer IgE directed against one or more antigens contained in the extract used in the ELISA, below the positivity threshold of the diagnostic ImmunoCAP assay.

Table 1 summarizes the mean AI values, standard deviation (SD), median, interquartile range (IQR), and 95% confidence intervals (CIs) for each group. GAA patients displayed significantly lower IgE avidity compared with CU− patients (*p* = 0.0368).

#### 3.1.2. Temporal Evolution of IgE Avidity

Longitudinal analysis was performed on sera from GAA patients at baseline (T = 0), three months (T = 1), and one year (T = 2) following diagnosis (Table A1 and Table 2).

A progressive increase in IgE avidity was observed over time, suggesting antibody affinity maturation during the course of infection.

In contrast, the CU+ group exhibited consistently high and stable IgE avidity values across all timepoints (T = 0, T = 1, T = 2), with no significant differences detected (Table A2 and Table A3).

### 3.2. Avidity of IgG4 Against Anisakis simplex Antigens

#### 3.2.1. IgG4 Avidity Index by ELISA: Total Larval vs. Excretory-Secretory (ES) Antigens

The AI of specific IgG4 was quantified by ELISA using sera from GAA, CU+, and CU− patients against both the total larval and excretory-secretory (ES) antigens of *A. simplex*. Only samples with IgG4 OD values ≥ 0.100 were analyzed; this threshold was defined as the mean OD of negative controls plus four standard deviations.

Table 3 presents the AI results for both antigens. No statistically significant differences were found among clinical groups (*p* > 0.05). All groups exhibited mean IgG4 AI values > 50%, consistent with the presence of high-avidity antibodies regardless of antigen type.

#### 3.2.2. Temporal Evolution of IgG4 Avidity

In GAA patients, the AI of IgG4 was evaluated at T = 0, T = 1, and T = 2 for both the total larval and ES antigens (Table 4, Table A4, Table A5, Table A6 and Table A7). A mild reduction in AI was detected at 3 months, followed by partial recovery at 1 year for both antigens, though sample sizes were limited.

In CU+ patients, variations in IgG4 avidity were also assessed (Table A5 and Table A8). A minor decrease was seen at 3 months for both total and ES antigens, with a tendency toward recovery or slight increase at 1 year for ES antigen.

#### 3.2.3. IgG4 Avidity by Western Blot

A total of 49 sera were analyzed by Western blot (23 GAA, 18 CU+, 8 CU−). Immunorecognition of protein bands at 15, 18, 37, 62.5, and >150 kDa was observed in 13.7% of samples, with partial recognition in the remaining sera.

**Figure 1:**
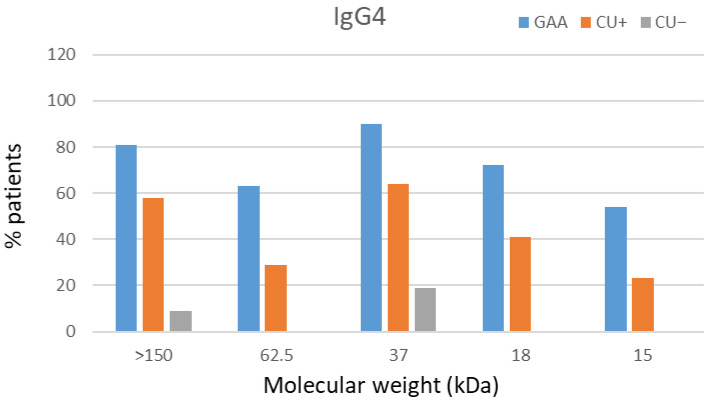
IgG4 immunorecognition of *Anisakis simplex* proteins. Frequency of IgG4-mediated band recognition in GAA, CU+, and CU− patient sera against CE antigen of *A. simplex*.

**Figure 2:**
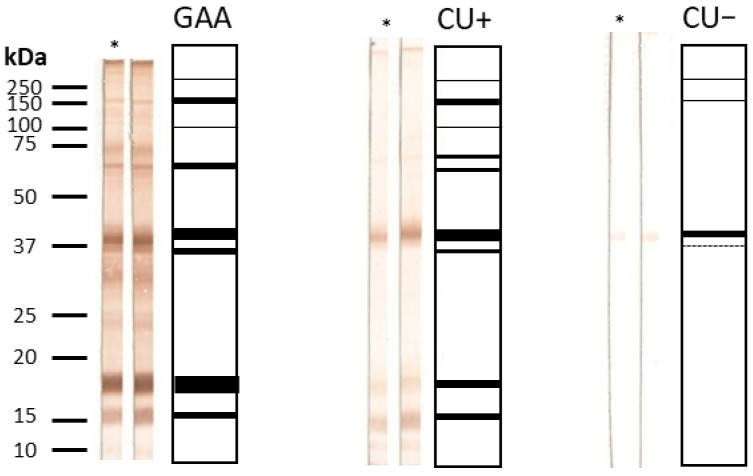
IgG4 Western blot summary: bands and avidity. Patterns of IgG4 band recognition and avidity indices in patient sera, highlighting complete and partial immunorecognition among groups; * indicates urea-treated strips.

The most complete banding profile (15–>150 kDa) was characteristic of GAA sera, whereas CU+ recognized primarily the 18–62.5 kDa range, and CU− displayed the narrowest pattern (18–37 kDa) (Figure 1 and Figure 2). No significant differences were observed in band profiles before and after urea treatment, supporting ELISA findings of uniformly high IgG4 avidity (>50%).

### 3.3. Avidity of IgG Against Anisakis simplex Total Larval and Excretory-Secretory (ES) Antigens

#### 3.3.1. Determination of IgG Avidity Index by ELISA

The IgG AI was determined by ELISA against both total larval and ES antigens. Only samples with OD values ≥ 2.702 (total antigen) or ≥0.914 (ES antigen) were included. Thresholds were established as the mean OD of negative controls plus four standard deviations.

Given the limited number of positive sera (three for total antigen), especially within CU+ and CU− groups, mean AI values were not representative. Individual GAA samples demonstrated avidity indices exceeding 50%, consistent with high-affinity antibodies (Table A9, Table A18 and Table A19).

#### 3.3.2. Temporal Evolution of IgG Avidity

Due to small sample size, temporal changes in IgG avidity could only be tracked in a few GAA and CU+ patients (Table A10, Table A11, Table A12, Table A13, Table A14, Table A15, Table A16 and Table A17). In GAA sera, AI remained consistently above 50% at all evaluated timepoints (baseline, 3 months, 1 year), indicating sustained high-avidity responses. The CU+ group showed similar trends, with no significant fluctuations.

#### 3.3.3. IgG Avidity by Western Blot

**Figure 3:**
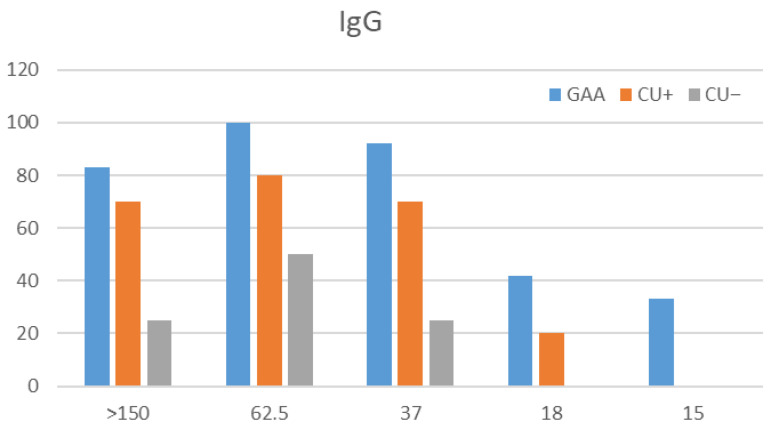
IgG immunorecognition of *Anisakis simplex* proteins. Frequency of IgG-mediated band recognition in GAA, CU+, and CU− patient sera against CE antigen of *A. simplex*.

**Figure 4:**
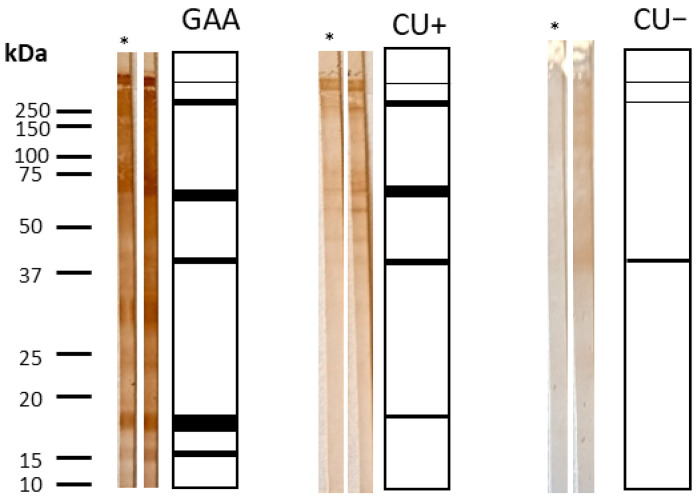
IgG Western blot summary: bands and avidity. Patterns of IgG band recognition and avidity indices in patient sera, highlighting complete and partial immunorecognition among groups; * indicates urea-treated strips.

Western blot analysis was performed on 49 sera (23 GAA, 18 CU+, 8 CU−) that met the inclusion criteria (OD ≥ 0.914). Immunorecognition was observed in 11.8% of sera, recognizing proteins at 15, 18, 37, 62.5, and >150 kDa. Partial recognition occurred in the remaining samples (Figure 3 and Figure 4).

No noticeable differences were observed between urea-treated and untreated membranes. The results mirrored ELISA findings, confirming that all GAA sera tested contained high-avidity IgG antibodies (>50%).

### 3.4. Avidity of IgA Against Anisakis simplex Total Larval and Excretory-Secretory (ES) Antigens

#### 3.4.1. IgA Avidity Index by ELISA

The IgA AI was determined by ELISA in sera from GAA, CU+, and CU− patients using total larval and ES antigens. Only sera with OD ≥ 1.615 (total antigen) or ≥1.271 (ES antigen) were included, thresholds established as the mean OD of negative controls plus four standard deviations.

#### 3.4.2. Temporal Evolution of IgA Avidity

In GAA patients, IgA AI values were evaluated at T = 0, T = 1, and T = 2 (Table A20, Table A23 and Table A24). A modest decline in AI was noted for the total antigen at one year, whereas avidity increased progressively for the ES antigen. These observations are preliminary given the limited sample size.

In the CU+ group, nonsignificant variations were observed (Table A21, Table A22, Table A25 and Table A26), reflecting stable or slightly decreasing IgA avidity over time.

#### 3.4.3. IgA Avidity by Western Blot

**Figure 5:**
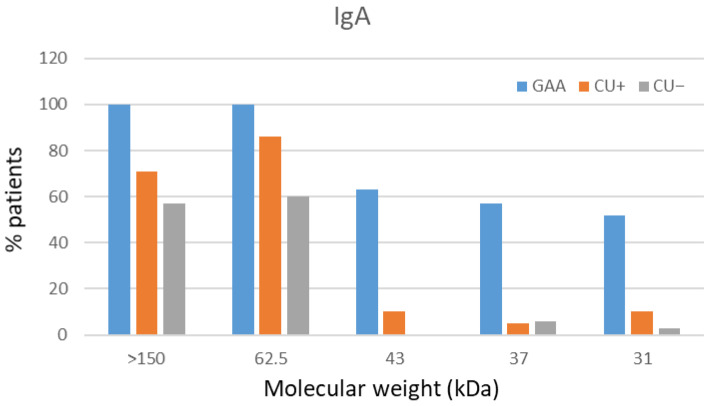
IgA immunorecognition of *Anisakis simplex* proteins. Frequency of IgA-mediated band recognition in GAA, CU+, and CU− patient sera against CE antigen of *A. simplex*.

**Figure 6:**
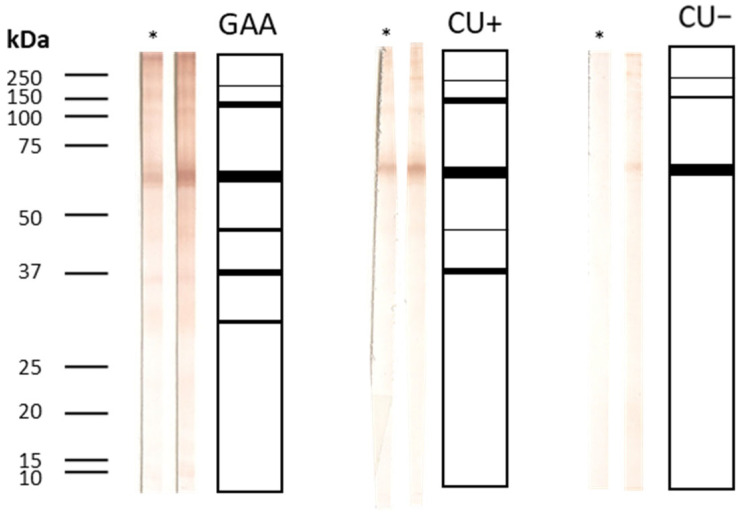
IgA Western blot summary: bands and avidity. Patterns of IgA band recognition and avidity indices in patient sera, highlighting complete and partial immunorecognition among groups; * indicates urea-treated strips.

Forty-nine sera (23 GAA, 18 CU+, 8 CU−) were subjected to Western blot analysis for IgA avidity. Only samples with mean OD ≥ 1.274 were included. Complete recognition of protein bands (31, 37, 43, 62.5, >150 kDa) was most frequent in GAA sera, with markedly weaker recognition in CU+ and CU− samples (Figure 5 and Figure 6).

Urea treatment caused no meaningful changes in the band profile, and all sera exhibited avidity values above 50%, consistent with the ELISA results.

## 4. Discussion

Previous studies have demonstrated the clinical importance of antibody avidity for the diagnosis and staging of parasitic diseases, particularly anisakiasis. Cuéllar et al. were the first to identify unique avidity profiles of specific immunoglobulins in patients with GAA compared to those with CU+, showing higher IgG avidity and lower IgE avidity in acute episodes, and further highlighting the impact of exposure history and dietary habits [9]. Building on these observations, González-Fernández et al. broadened the scope by underscoring the diagnostic value of non-IgE antibodies, most notably IgG4 and IgA, and proposed that measuring both their levels can improve differentiation among acute, chronic, and sensitized patient groups [4]. Elefant et al. contributed empirical evidence supporting the use of IgG avidity for staging chronic parasite infections and recommended the extension of avidity analyses to other immunoglobulin classes [5]. In line with this, Kaneva et al. validated the relevance of non-IgE antibody avidity in distinguishing acute from chronic parasitic diseases [7]. Collectively, these works support antibody avidity, especially for IgE, IgG4, and IgG, as an effective biomarker for disease stage, prognosis, and improved clinical management of *A. simplex*-associated allergic conditions.

Longitudinal analysis by Elefant et al. in patients treated for toxocariosis revealed that IgE levels decreased significantly within the first year post-treatment, IgA declined during the second year, while IgG levels diminished only from the fourth year onward. All patients displayed persistently high IgG avidity, confirming chronic infection [5]. These results demonstrate IgG avidity as a marker of chronicity and align with current findings on non-IgE antibody avidity. The urea-based dissociative ELISA methodology employed in these studies directly validates the utility of avidity assessment in staging parasitic infections.

Experimental work by Cho et al. indicated that reinfection with *A. simplex* larvae provokes a more rapid and intense allergic response in rats, reflected in increased antibody levels and heightened allergic manifestations, yet the severity of clinically apparent allergy was not explained solely by specific IgE avidity [13]. Their findings suggest that reinfection is pivotal in the development and amplification of allergic responses, implicating factors beyond IgE avidity, such as elevated IgM, in determining allergic outcomes.

The present study sought to further assess avidity as a diagnostic marker by identifying specific *A. simplex* antigens involved in affinity maturation and evaluating longitudinal changes in antibody avidity from initial infection through subsequent exposures. IgG4 is frequently associated with immune tolerance and blocking activity in allergic reactions. Measuring the avidity of anti-*Anisakis* IgG4 could help to determine whether high-avidity IgG4 facilitates the suppression of allergic symptoms by competing with IgE for antigen binding, thereby reducing mast cell degranulation and hypersensitivity.

Our findings demonstrate that IgG4 antibodies generated against both total larval and ES antigens exhibit uniformly high avidity (>50%) across all patient groups. No statistically significant differences in IgG4 avidity were found between GAA, CU+, and CU− patients for either antigen type. A transient decline in IgG4 avidity was observed in GAA patients at three months, with partial recovery detected at one year. These longitudinal observations suggest that monitoring IgG4 avidity could provide a useful tool to differentiate acute from chronic exposure. While acute GAA shows transient fluctuations in IgG4 avidity, chronic or repeated exposures tend to maintain or progressively increase IgG4 avidity, reflecting sustained antigenic stimulation. This pattern may offer translational relevance for patient stratification and immune monitoring in parasitic infections. Conversely, IgE avidity increased progressively over time in the GAA cohort. CU+ patients displayed persistently high IgE avidity throughout follow-up. High avidity of *Anisakis*-specific IgE can coexist with comparatively low IgE concentrations, reflecting a mature, and high-affinity antibody response despite lower overall antibody levels. For IgG4 (both total antigen and ES antigen), a modest decrease in avidity was noted at three months in CU+ patients, with a trend toward recovery or increase at one year in ES antigen-specific responses. Western blot analysis confirmed high-avidity IgG4 in all clinical groups, with no significant band recognition changes after urea treatment. Broader antigenic band recognition by high-avidity IgG4 was observed in GAA patients when compared to CU+ and CU−. High-avidity IgG4 may denote a more mature and regulated immune response, particularly in chronically exposed individuals, and could help separate clinically relevant allergy from mere sensitization. However, sera from various patient groups showed little change in antigen recognition upon urea treatment, corroborating the quantitative ELISA findings.

Specifically, recognition of the complete panel of protein bands (15 kDa, 18 kDa, 37 kDa, 62.5 kDa, and >150 kDa) was seen in seven GAA patient samples (13.72%), while six GAA sera (11.76%) demonstrated full recognition in the IgG avidity test, and seven (13.72%) in the IgG4 avidity assessment. The comparatively low number of IgG-positive sera, especially outside the GAA group, limits the generalizability of these observations. Nonetheless, GAA patients consistently showed high-avidity IgG for both total larval and ES antigens. Western blot analysis complemented the ELISA findings by confirming high-avidity IgG and broad antigenic recognition in GAA cases.

Regarding IgA avidity, a slight decrease in AI was detected for total larval antigens, while AI for ES antigens increased, but the limited sample size prevents definitive conclusions. IgA, central to mucosal defense in the gastrointestinal tract where *Anisakis* infection occurs, may confer protection against larval invasion or chronic sensitization. High-avidity IgA could reflect more efficient neutralization or elimination of the parasite at mucosal surfaces, potentially mitigating acute and chronic allergic manifestations. Importantly, only three GAA patients (5.88%) showed full immune recognition of the panel of protein bands (31 kDa, 37 kDa, 43 kDa, 62.5 kDa, and >150 kDa) in IgA assays; in other samples, recognition was partial. These findings align with quantitative ELISA avidity results indicating generally high IgA avidity (>50%). The low prevalence of full-antigen recognition is consistent with the overall immune response profile. Western blot further validated the presence of high-avidity IgA in both GAA and CU+ patients, with most sera displaying only partial recognition of antigenic bands; complete band recognition was rare. Thus, while IgA avidity is generally high in GAA, it may be less effective than IgE or IgG4 avidity for differentiating between clinical groups, but still adds richness to the serological landscape. This is the first longitudinal assessment of IgA avidity for total larval and ES antigens in GAA and CU+ patients, though trends remain inconclusive due to the limited sample numbers. Notably, Kaneva et al. described the limited utility of IgA for distinguishing acute from chronic infection [7].

Typically, antibody avidity increases from low levels in acute infection to higher levels in chronic infection in other parasitic scenarios. In the case of anisakiasis, even if there are possible chronic immunologically mediated manifestations after acute parasitic contact, in humans there does not exist a chronic infection, but antigenic stimulation could be elicited by *Anisakis* antigen containing fish intake. By analyzing the progression of IgG4, IgA, and IgG avidity, our study demonstrates that a combined evaluation of these indices may improve the diagnosis of anisakiasis and reduce misclassification compared to single-antibody measurement approaches. Furthermore, non-IgE antibodies, particularly IgG4 and IgA, may provide complementary information regarding clinical severity and immune regulation during repeated exposures. While IgE responses are associated with immediate hypersensitivity, the stable or increasing avidity of IgG4 and IgA could reflect cumulative antigenic stimulation and immune control, potentially serving as more reliable markers for predicting clinical outcomes in anisakiasis. Incorporating these antibody classes into longitudinal monitoring may enhance patient stratification and risk assessment. Fenoy et al. found a significant rise in antibody avidity associated with chronic toxocariasis, supporting avidity as a marker of infection stage [14]. In our cohort, high-avidity IgG4 was consistently identified by both ELISA and Western blot, while low IgA avidity predominated in acute GAA cases. Overall avidity indices showed slight declines over time, particularly in CU+ patients, reflecting dynamic antibody maturation and substantiating the use of avidity for temporal infection profiling.

For other human parasitic diseases, antibody avidity is characteristically lower during early infection and increases with chronic exposure, as established in toxoplasmosis [15] and toxocariasis [14]. Although only a subset of sera was followed longitudinally, the trend toward increasing avidity was evident. Detection of high-avidity IgG4 antibodies in chronic *A. simplex* infection confirms that affinity maturation serves as a marker of prolonged exposure to *Anisakis* antigens and immune regulation, consistent with other parasitic conditions.

Methodological considerations such as serum dilution optimization are critical to accommodate individual variations in immunoglobulin levels and cross-reactivity, and to ensure standardization and reproducibility of avidity assays [12,16]. Commercial assays (e.g., VIDAS Toxo IgG avidity) recommend targeting antibody concentrations around 15 U/mL for reliable assessment [17]. Future research should aim to individualize serum dilutions and build standardized protocols for precise IgG4 avidity evaluation as a marker of chronicity in *A. simplex* allergy.

Limitations of this study include its modest sample size, recruitment from a single center, and predominantly cross-sectional design, all of which restrict generalizability and the interpretation of temporal trends. Potential confounders such as co-sensitizations, dietary variations, and technical variability in serological assays were not fully controlled and may have influenced results. Prospective studies should incorporate larger, more diverse populations, employ longitudinal designs, and rigorously address confounding factors. Establishing correlations between serological findings and clinical endpoints, and integrating further immunological markers such as cytokine profiles or cellular responses, will deepen our understanding of anisakiasis pathogenesis and enhance the accuracy of diagnosis and disease prognosis.

In conclusion, our data reveal that avidity indices for specific immunoglobulins, particularly IgE and IgG4, effectively distinguish between the acute form of GAA and CU+. GAA patients display a lower IgE and higher IgG4 avidity than CU+ subjects, reflecting distinct immune responses. Antibody avidity measurement enhances the specificity of serological tests and enables more precise differentiation between recent and past exposure. To facilitate comparison and highlight the diagnostic and prognostic relevance of each antibody class, the key findings on avidity are summarized in Table 5. These findings support the role of antibody avidity as a valuable biomarker for disease staging and clinical management, indicating that tracking antibody maturation may improve prognosis and facilitate personalized treatment strategies in *A. simplex*-associated allergic disease.

## 5. Conclusions

This study demonstrates that avidity indices of specific immunoglobulins, particularly IgE and IgG4, enable reliable differentiation between acute gastroallergic anisakiasis and chronic urticaria sensitized to *Anisakis simplex*. GAA patients consistently present lower IgE avidity and higher IgG4 avidity compared to CU+ subjects, highlighting the diagnostic utility of antibody avidity profiles for distinguishing disease stage and immune phenotype. Measurement of immunoglobulin avidity, beyond antibody titers, enhances serological specificity, facilitates the identification of current versus past exposure, and supports individualized risk assessment for severe allergic reactions. Moreover, elevated IgG4 and IgG avidity in acute cases suggests a potential regulatory or protective role for IgG4, while IgA avidity does not contribute substantially to clinical discrimination between phenotypes. Overall, assessment of antibody maturation and avidity provides valuable biomarkers for guiding diagnosis, staging, and management in *A. simplex*-associated allergic diseases.

## Figures and Tables

**Table 1:** Avidity indices of serum IgE in clinical groups. Mean avidity index (%), standard deviation, median, interquartile range, and 95% confidence interval of specific IgE against *A. simplex* total larval antigen, presented for GAA, CU+, and CU− cohorts.

Group	n	Mean AI (%)	SD	Median (IQR)	95% CI
GAA	22	79.86	11.81	77.33 (70.37–87.75)	74.62–85.09
CU+	22	88.50	16.28	85.14 (76.81–97.34)	81.28–95.71
CU−	21	92.02	21.26	90.09 (81.43–104.20)	82.34–101.70

**Table 2:** Temporal evolution of IgE avidity in GAA patients. Avidity index (%) of specific IgE at baseline, three months, and one year in GAA patient sera against total larval antigen.

Timepoint	n	Mean AI (%)	SD	Median (IQR)
T = 0	11	75.12	9.54	74.09 (68.81–85.84)
T = 1	11	81.06	9.99	80.36 (73.35–85.35)
T = 2	4	99.93	11.24	102.70 (88.12–109.00)

**Table 3:** IgG4 avidity index by antigen and clinical group. Mean avidity index (%) of serum IgG4 against total larval and excretory-secretory (ES) antigens of *A. simplex* in GAA, CU+, and CU− patient groups.

Group	n (Total)	Mean AI (%) Total	SD	Median (IQR)	n (ES)	Mean AI (%) ES	SD	Median (IQR)
GAA	10	69.08	15.09	63.91 (60.15–78.64)	13	67.52	25.66	71.48 (48.94–93.81)
CU+	7	63.89	10.58	67.50 (53.76–74.16)	10	55.88	14.27	52.63 (43.52–64.35)
CU−	10	62.38	10.17	63.20 (52.51–71.21)	5	58.49	12.50	61.18 (46.26–69.37)

GAA: Gastroalergic Anisakiasis; CU+: Chronic Urticaria, *Anisakis* positive; CU−: Chronic Urticaria, *Anisakis* negative; SD: Standard Deviation; IQR: Interquartile Range.

**Table 4:** Temporal evolution of IgG4 avidity in GAA patients. Avidity index (%) of specific IgG4 in GAA patients tracked longitudinally for total larval antigen and ES antigen.

Antigen Type	Timepoint	n	Mean AI (%)	SD	Median (IQR)
Total Larval	T = 0	5	72.19	17.10	61.63 (60.58–77.35)
	T = 1	5	53.08	20.47	44.87 (31.97–65.63)
	T = 2	2	58.73	7.54	58.73 (53.41–64.05)
ES	T = 0	8	73.99	28.51	82.19 (49.61–96.70)
	T = 1	8	66.90	21.46	70.77 (45.89–85.01)
	T = 2	4	65.81	24.18	68.62 (41.46–87.54)

**Table 5:** Diagnostic and prognostic relevance of avidity antibodies.

Antibody	Acute/GAA	Chronic/CU+	Prognostic Relevance
IgE	AGA < UC+	High maintained	Immediate hypersensitivity
IgG4	AGA > UC+	Stable and progressive increase	Differentiates acute vs. chronic exposure; reflects immune tolerance
IgA	Low	Maintained	Mucosal immunity

## Data Availability

The original contributions presented in this study are included in the article/Supplementary Material. Further inquiries can be directed to the corresponding authors.

## Supplementary Materials

The following supporting information can be downloaded at: https://www.mdpi.com/article/10.3390/antib15010013/s1, Figure S1: Western-blot; Table S1: Data.

## Appendix A

**Table A1:** Temporal Avidity Index of IgE in GAA. Avidity index (%) of specific IgE against *A. simplex* total larval antigen over baseline, 3 months, and 1 year in gastro-allergic anisakiasis (GAA) patient sera.

Serum	Group	Age	Sex	T = 0	T = 1	T = 2
2	GAA	65	F	87.35	72.59	84.46
5	GAA	68	F	79.50	80.36	109.83
24	GAA	60	F	70.49	97.18	106.32
50	GAA	46	M	68.65	81.05	99.13
62	GAA	40	F	74.09	80.00	nd
64	GAA	64	M	74.89	65.63	nd
69	GAA	51	F	85.84	75.73	nd
71	GAA	75	M	88.97	81.42	nd
77	GAA	58	M	70.03	98.96	nd
78	GAA	52	F	57.67	85.35	nd
79	GAA	52	F	68.81	73.35	nd

F: Female, M: Male, nd: not determined.

**Table A2:** Temporal Avidity Index of IgE in CU+. Avidity index (%) of specific IgE against *A. simplex* total larval antigen at baseline, 3 months, and 1 year in chronic urticaria patients with *Anisakis* sensitization (CU+).

Serum	Group	Age	Sex	T = 0	T = 1	T = 2
9	CU+	28	M	96.58	93.52	90.87
13	CU+	74	M	72.22	nd	95.60
15	CU+	52	M	71.86	95.90	82.40
21	CU+	63	M	131.07	nd	111.89
22	CU+	29	M	82.09	92.79	nd
23	CU+	51	M	93.36	nd	97.74
26	CU+	73	M	89.74	60.49	94.52
28	CU+	73	M	82.76	104.33	nd
29	CU+	70	M	86.75	68.37	107.11
30	CU+	43	M	111.75	99.13	78.16
33	CU+	51	M	66.91	62.14	86.15
38	CU+	25	M	76.49	66.82	64.84
140	CU+	65	M	nd	83.33	80.98
49	CU+	34	M	80.78	54.37	76.87
53	CU+	66	M	68.47	96.79	95.93
57	CU+	70	M	84.87	80.00	nd
66	CU+	60	M	76.91	110.66	106.63
70	CU+	70	M	80.30	nd	92.63
74	CU+	39	M	117.34	nd	105.00
76	CU+	54	M	99.61	nd	92.05
91	CU+	70	M	88.92	96.57	nd
98	CU+	46	M	103.71	114.89	nd

CU+: Chronic Urticaria, *Anisakis* positive; T = 0: Baseline; T = 1: Three months; T = 2: One year; M: Male; nd: Not determined.

**Table A3:** Statistical Summary of IgE Avidity in CU+. Summary statistics for specific IgE avidity index (%) against *A. simplex* total larval antigen in CU+ patients at baseline, 3 months, and 1 year.

Parameter	CU+ (T = 0)	CU+ (T = 1)	CU+ (T = 2)
Mean	88.65	86.26	91.73
SD	16.66	18.84	12.45
Median	84.87	93.15	92.63
IQR	76.70–98.10	67.20–98.55	81.69–101.40
95% CI	81.06–96.23	76.22–96.29	85.32–98.13
n	21	16	17

CU+: Chronic Urticaria, *Anisakis* positive; T = 0: Baseline; T = 1: Three months; T = 2: One year; SD: Standard Deviation; IQR: Interquartile Range; CI: Confidence Interval.

**Table A4:** Temporal Avidity Index of IgG4 in GAA. Avidity index (%) of specific IgG4 against *A. simplex* total larval antigen at baseline, 3 months, and 1 year in GAA patient sera.

Serum	Group	Age	Sex	T = 0	T = 1	T = 2
2	GAA	65	M	77.35	nd	53.41
3	GAA	38	M	100.00	51.55	nd
5	GAA	68	M	61.41	31.97	64.05
24	GAA	60	M	60.58	35.34	nd
50	GAA	46	F	61.63	44.87	nd

GAA: Gastroalergic Anisakiasis; T = 0: Baseline; T = 1: Three months; T = 2: One year; M: Male; F: Female; nd: Not determined.

**Table A5:** Temporal Avidity Index of IgG4 in CU+. Avidity index (%) of specific IgG4 against *A. simplex* total larval antigen at baseline and 3 months in CU+ patient sera.

Serum	Group	Age	Sex	T = 0	T = 1
29	CU+	70	M	67.50	37.50
38	CU+	25	M	68.32	48.85
98	CU+	46	M	74.16	79.39

CU+: Chronic Urticaria, *Anisakis* positive; T = 0: Baseline; T = 1: Three months; M: Male.

**Table A6:** Temporal Avidity Index of IgG4 (ES antigen) in GAA. Avidity index (%) of specific IgG4 against *A. simplex* excretory-secretory antigen at baseline, 3 months, and 1 year in GAA patient sera.

Serum	Group	Age	Sex	T = 0	T = 1	T = 2
2	GAA	65	M	72.00	68.22	75.93
3	GAA	38	M	95.24	62.88	nd
5	GAA	68	M	42.32	40.21	34.58
24	GAA	60	M	92.37	91.46	91.41
50	GAA	46	F	71.47	73.32	61.30
62	GAA	40	M	21.97	31.35	nd
64	GAA	64	M	99.32	86.16	nd
71	GAA	75	M	97.17	81.54	nd

GAA: Gastroalergic Anisakiasis; T = 0: Baseline; T = 1: Three months; T = 2: One year; M: Male; F: Female; nd: Not determined.

**Table A7:** Statistical Summary of IgG4 Avidity (ES Antigen) in GAA. Summary statistics for specific IgG4 avidity index (%) against ES antigen in GAA patient sera at baseline, 3 months, and 1 year.

Parameter	GAA (T = 0)	GAA (T = 1)	GAA (T = 2)
Mean	73.99	66.90	65.81
SD	28.51	21.46	24.18
Median	82.19	70.77	68.62
IQR	49.61–96.70	45.89–85.01	41.46–87.54
95% CI	50.16–97.82	48.96–84.84	27.34–104.30
n	8	8	4

GAA: Gastroalergic Anisakiasis; T = 0: Baseline; T = 1: Three months; T = 2: One year; SD: Standard Deviation; IQR: Interquartile Range; CI: Confidence Interval.

**Table A8:** Temporal Avidity Index of IgG4 (ES antigen) in CU+. Avidity index (%) of specific IgG4 against *A. simplex* ES antigen at baseline, 3 months, and 1 year in CU+ patient sera.

Serum	Group	Age	Sex	T = 0	T = 1	T = 2
29	CU+	70	M	52.24	43.11	62.40
33	CU+	51	M	43.92	nd	53.70
38	CU+	25	M	64.80	60.59	46.68
57	CU+	70	M	61.95	43.82	nd
66	CU+	60	M	50.76	51.17	88.69
74	CU+	39	M	42.37	43.11	nd

CU+: Chronic Urticaria, *Anisakis* positive; T = 0: Baseline; T = 1: Three months; T = 2: One year; M: Male; nd: Not determined.

**Table A9:** Mean IgG Avidity Index for ES Antigen. Mean avidity index (%) of specific IgG against ES antigen by clinical group and pathology.

Parameter	GAA	CU+	CU−
Mean	78.20	71.80	73.16
SD	12.20	13.18	4.85
Median	80.37	69.87	73.16
IQR	71.58–86.91	62.07–84.95	69.73–76.59
95% CI	68.83–87.58	60.78–82.82	29.56–116.70
n	9	8	2

GAA: Gastroalergic Anisakiasis; CU+: Chronic Urticaria, *Anisakis* positive; CU−: Chronic Urticaria, *Anisakis* negative; SD: Standard Deviation; IQR: Interquartile Range; CI: Confidence Interval.

**Table A10:** Temporal Avidity Index of IgG in GAA. Avidity index (%) of specific IgG against total larval antigen at baseline and 3 months in GAA patient sera.

Serum	Group	Age	Sex	T = 0	T = 1
3	GAA	38	M	68.28	65.63
5	GAA	68	M	55.43	44.30
24	GAA	60	M	58.18	63.19
32	GAA	36	F	24.36	26.12
35	GAA	35	M	47.03	50.94

GAA: Gastroalergic Anisakiasis; T = 0: Baseline; T = 1: Three months; M: Male; F: Female.

**Table A11:** Statistical Summary of IgG Avidity in GAA. Summary statistics for specific IgG avidity index (%) against total larval antigen at baseline and 3 months in GAA patients.

Parameter	GAA (T = 0)	GAA (T = 1)
Mean	50.66	50.04
SD	16.54	15.98
Median	55.43	50.94
IQR	35.70–63.23	35.21–64.41
95% CI	30.12–71.20	30.19–69.88
n	5	5

GAA: Gastroalergic Anisakiasis; T = 0: Baseline; T = 1: Three months; SD: Standard Deviation; IQR: Interquartile Range; CI: Confidence Interval.

**Table A12:** Temporal Avidity Index of IgG in CU+. Avidity index (%) of specific IgG against total larval antigen at baseline and 3 months in CU+ patient sera.

Serum	Group	Age	Sex	T = 0	T = 1
9	CU+	28	M	58.00	66.09
13	CU+	74	M	49.78	43.75
21	CU+	63	M	38.64	40.52
22	CU+	29	M	26.92	32.84
26	CU+	73	F	39.12	29.26
28	CU+	73	F	56.78	47.53
30	CU+	43	M	49.01	43.66
38	CU+	25	M	71.96	69.84
40	CU+	65	M	32.14	39.71
46	CU+	65	M	23.70	38.55
53	CU+	66	F	36.08	40.63

CU+: Chronic Urticaria, *Anisakis* positive; T = 0: Baseline; T = 1: Three months; M: Male; F: Female.

**Table A13:** Statistical Summary of IgG Avidity in CU+. Summary statistics for specific IgG avidity index (%) against total larval antigen in CU+ patients at baseline and 3 months.

Parameter	CU+ (T = 0)	CU+ (T = 1)
Mean	43.83	44.76
SD	14.69	12.55
Median	39.12	40.63
IQR	32.14–56.78	38.55–47.53
95% CI	33.96–53.70	36.33–53.19
n	11	11

CU+: Chronic Urticaria, Anisakis positive; T = 0: Baseline; T = 1: Three months; SD: Standard Deviation; IQR: Interquartile Range; CI: Confidence Interval.

**Table A14:** Longitudinal IgG Avidity Index in GAA. Avidity index (%) of specific IgG against total larval antigen at baseline, 3 months, and 1 year in GAA patient sera.

Serum	Group	Age	Sex	T = 0	T = 1	T = 2
2	GAA	65	M	81.66	73.13	68.96
5	GAA	68	M	nd	66.40	54.44
24	GAA	60	M	80.37	99.26	116.01
50	GAA	46	F	87.20	87.71	nd
64	GAA	64	M	86.61	88.56	nd
69	GAA	51	F	69.87	77.47	nd

GAA: Gastroalergic Anisakiasis; T = 0: Baseline; T = 1: Three months; T = 2: One year; M: Male; F: Female; nd: Not determined.

**Table A15:** Statistical Summary of IgG Avidity (ES antigen) in GAA. Summary statistics for specific IgG avidity index (%) against ES antigen in GAA patients over time.

Parameter	GAA (T = 0)	GAA (T = 1)	GAA (T = 2)
Mean	81.15	82.09	82.09
SD	6.98	11.97	11.97
Median	81.66	82.60	68.96
IQR	75.12–86.91	71.45–91.24	54.45–116.00
95% CI	72.49–89.81	69.54–94.65	−0.14–159.80
n	5	6	3

GAA: Gastroalergic Anisakiasis; T = 0: Baseline; T = 1: Three months; T = 2: One year: SD: Standard Deviation; IQR: Interquartile Range; CI: Confidence Interval.

**Table A16:** Longitudinal IgG Avidity Index in CU+. Avidity index (%) of specific IgG against total larval antigen at baseline, 3 months, and 1 year in CU+ patient sera.

Serum	Group	Age	Sex	T = 0	T = 1	T = 2
13	CU+	74	M	72.38	nd	46.99
29	CU+	70	M	82.23	93.97	91.67
38	CU+	25	M	85.85	111.47	83.66
57	CU+	70	M	89.37	95.40	nd
66	CU+	60	M	67.34	75.31	nd

CU+: Chronic Urticaria, *Anisakis* positive; T = 0: Baseline; T = 1: Three months; T = 2: One year; M: Male; nd: Not determined.

**Table A17:** Statistical Summary of IgG Avidity (ES antigen) in CU+. Summary statistics for specific IgG avidity index (%) against ES antigen in CU+ patients over baseline, 3 months, and 1 year.

Parameter	CU+ (T = 0)	CU+ (T = 1)	CU+ (T = 2)
Mean	79.44	94.04	74.11
SD	9.27	14.79	23.82
Median	82.24	94.69	83.66
IQR	69.87–87.61	79.98–107.50	47.00–91.67
95% CI	67.93–90.95	70.50–117.60	14.94–133.30
n	5	4	3

CU+: Chronic Urticaria, *Anisakis* positive; T = 0: Baseline; T = 1: Three months; T = 2: One year; SD: Standard Deviation; IQR: Interquartile Range; CI: Confidence Interval.

**Table A18:** Mean IgG Avidity Index by Pathology. Mean avidity index (%) of specific IgG against total larval antigen by clinical pathology groups.

Parameter	GAA	CU+	CU−
Mean (X) (%)	61.88	78.88	85.05
SD	14.52	14.32	14.29
Median	53.36	84.64	82.97
IQR	50.62–74.63	67.13–87.97	72.88–99.30
95% CI	49.74–74.02	63.85–93.90	62.31–107.80
n	8	6	4

GAA: Gastroalergic Anisakiasis; CU+: Chronic Urticaria, *Anisakis* positive; CU−: Chronic Urticaria, *Anisakis* negative; SD: Standard Deviation; IQR: Interquartile Range; CI: Confidence Interval.

**Table A19:** Mean IgG Avidity Index for ES Antigen by Pathology. Mean avidity index (%) of specific IgG against ES antigen by pathology group.

Parameter	GAA	CU+	CU−
Mean (X) (%)	49.04	56.05	49.10
SD	14.80	12.43	8.49
Median	49.71	58.23	46.62
IQR	41.51–59.59	49.18–64.08	42.12–58.55
95% CI	38.46–59.63	45.65–66.44	28.00–70.19
n	10	8	3

GAA: Gastroalergic Anisakiasis; CU+: Chronic Urticaria, *Anisakis* positive; CU−: Chronic Urticaria, *Anisakis* negative; SD: Standard Deviation; IQR: Interquartile Range; CI: Confidence Interval.

**Table A20:** Temporal IgA Avidity Index in GAA. Avidity index (%) of specific IgA against total larval antigen at baseline, 3 months, and 1 year in GAA patient sera.

Serum	Group	Age	Sex	T = 0	T = 1	T = 2
2	GAA	65	M	52.58	nd	35.05
5	GAA	68	M	54.14	nd	40.76
24	GAA	60	M	57.63	49.48	nd
32	GAA	36	F	75.35	61.05	nd

GAA: Gastroalergic Anisakiasis; T = 0: Baseline; T = 1: Three months; T = 2: One year; M: Male; F: Female; nd: Not determined.

**Table A21:** Temporal IgA Avidity Index in CU+. Avidity index (%) of specific IgA against total larval antigen at baseline, 3 months, and 1 year in CU+ patient sera.

Serum	Group	Age	Sex	T = 0	T = 1	T = 2
9	CU+	28	M	71.79	nd	39.23
13	CU+	74	M	53.15	nd	25.60
15	CU+	52	F	85.33	nd	47.56
21	CU+	63	M	83.94	nd	38.76
22	CU+	29	M	48.63	88.67	nd
23	CU+	51	M	107.58	61.64	nd
30	CU+	43	M	78.97	72.91	nd
33	CU+	51	M	86.41	44.70	nd
38	CU+	25	M	99.72	69.18	nd
40	CU+	65	M	70.24	48.35	nd
46	CU+	65	M	93.44	69.58	43.90
149	CU+	34	F	nd	36.34	40.26

CU+: Chronic Urticaria, *Anisakis* positive; T = 0: Baseline; T = 1: Three months; T = 2: One year; M: Male; F: Female; nd: Not determined.

**Table A22:** Statistical Summary of IgA Avidity in CU+. Summary statistics for specific IgA avidity index (%) against total larval antigen in CU+ patients over baseline, 3 months, and 1 year.

Parameter	CU+ (T = 0)	CU+ (T = 1)	CU+ (T = 2)
Mean	79.93	61.42	39.22
SD	18.12	17.24	7.46
Median	83.94	65.41	39.75
IQR	70.24–93.44	45.61–72.08	35.47–44.82
95% CI	67.75–92.10	47.01–75.84	31.39–47.05
n	11	8	6

CU+: Chronic Urticaria, *Anisakis* positive; T = 0: Baseline; T = 1: Three months; T = 2: One year; SD: Standard Deviation; IQR: Interquartile Range; CI: Confidence Interval.

**Table A23:** Temporal IgA Avidity Index for ES Antigen in GAA. Avidity index (%) of specific IgA against ES antigen at baseline, 3 months, and 1 year in GAA patient sera.

Serum	Group	Age	Sex	T = 0	T = 1	T = 2
2	GAA	65	M	19.87	32.39	nd
5	GAA	68	M	59.30	50.91	49.40
24	GAA	60	M	52.86	49.10	nd
62	GAA	40	M	33.59	68.21	nd
64	GAA	64	M	50.46	48.04	nd

GAA: Gastroalergic Anisakiasis; T = 0: Baseline; T = 1: Three months; T = 2: One year.

**Table A24:** Statistical Summary of IgA Avidity (ES antigen) in GAA. Summary statistics for specific IgA avidity index (%) against ES antigen in GAA patients at baseline and 3 months.

Parameter	GAA (T = 0)	GAA (T = 1)
Mean	43.22	49.73
SD	16.14	12.72
Median	50.46	49.10
IQR	26.74–56.09	40.22–59.56
95% CI	23.18–63.26	33.94–65.52
n	5	5

GAA: Gastroalergic Anisakiasis; T = 0: Baseline; T = 1: Three months; SD: Standard Deviation; IQR: Interquartile Range; CI: Confidence Interval.

**Table A25:** Temporal IgA Avidity Index for ES Antigen in CU+. Avidity index (%) of specific IgA against ES antigen at baseline, 3 months, and 1 year in CU+ patient sera.

Serum	Group	Age	Sex	T = 0	T = 1	T = 2
9	CU+	28	M	64.76	66.35	61.05
13	CU+	74	M	31.66	19.69	50.21
21	CU+	63	M	57.73	45.80	54.55
29	CU+	70	M	58.72	nd	62.40
49	CU+	34	F	48.14	71.01	nd

CU+: Chronic Urticaria, *Anisakis* positive; T = 0: Baseline; T = 1: Three months; T = 2: One year; M: Male; F: Female; nd: Not determined.

**Table A26:** Statistical Summary of IgA Avidity (ES antigen) in CU+. Summary statistics for specific IgA avidity index (%) against ES antigen in CU+ patients at baseline, 3 months, and 1 year.

Parameter	CU+ (T = 0)	CU+ (T = 1)	CU+ (T = 2)
Mean	52.21	50.72	57.06
SD	12.94	23.40	5.71
Median	57.74	56.08	57.80
IQR	39.90–61.74	26.22–69.84	51.30–62.07
95% CI	36.15–68.27	13.48–87.95	47.98–66.14
n	5	4	4

CU+: Chronic Urticaria, *Anisakis* positive; T = 0: Baseline; T = 1: Three months; T = 2: One year; SD: Standard Deviation; IQR: Interquartile Range; CI: Confidence Interval.
