# Supplementary material for: Antibody Avidity Profiles as Diagnostic Biomarkers in Differentiating Acute and Chronic Anisakis simplex—Related Allergic Diseases
Source: Antibodies (Basel). 2026 Feb 6;15(1):13. doi: 10.3390/antib15010013 (PMC12921752; doi:10.3390/antib15010013)
Supplement: Supplementary file 1 [file antibodies-15-00013-s001.zip › Figure S1 - Western blot.pdf]

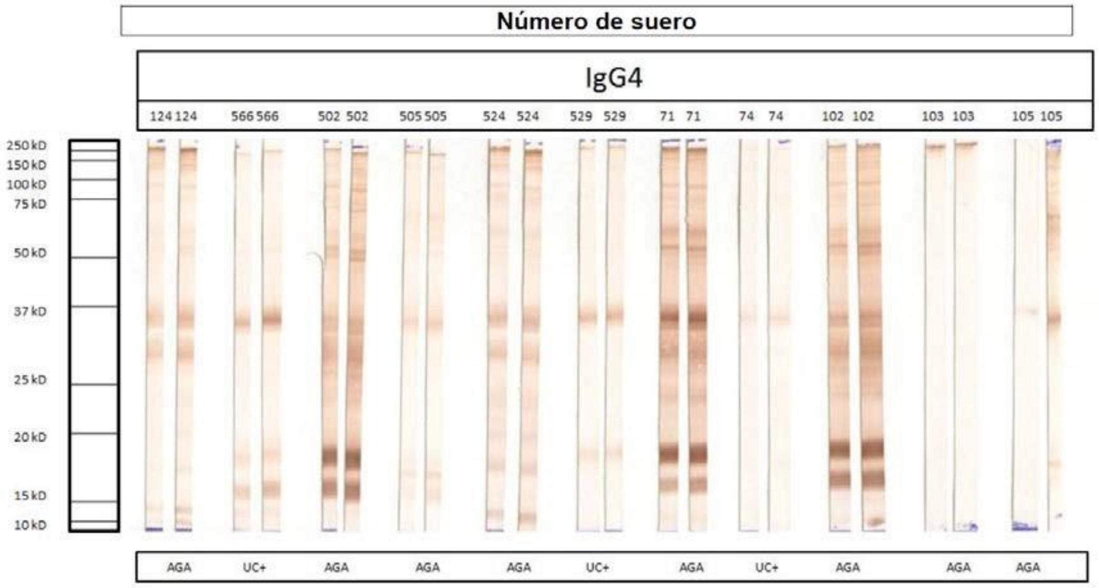

Figure S1A. Avidity of specific IgG4 in the sera studied (GAA, CU+, CU-, and Ctr). The nitrocellulose strip on the left was treated with urea.

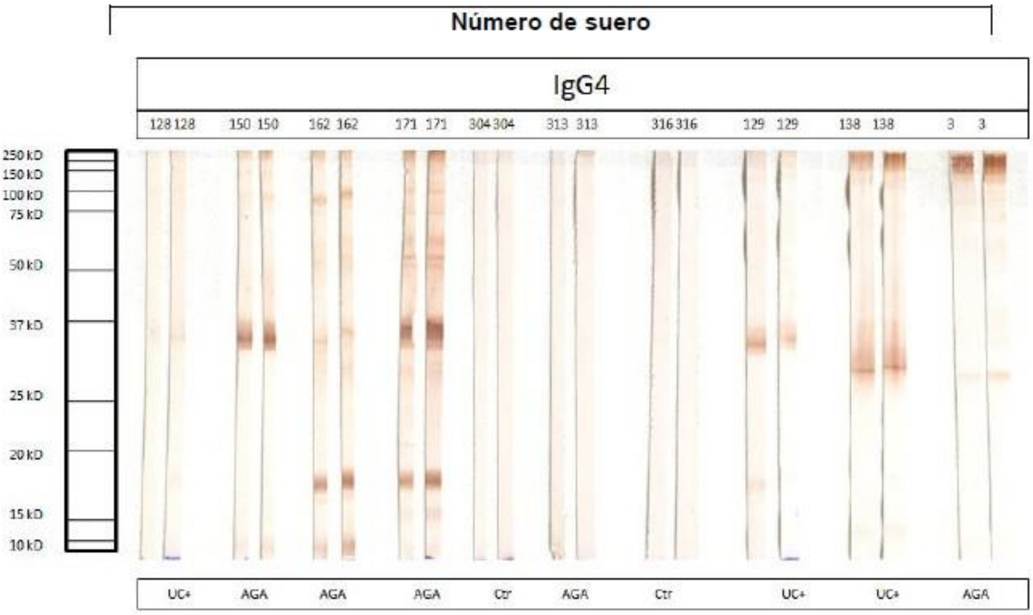

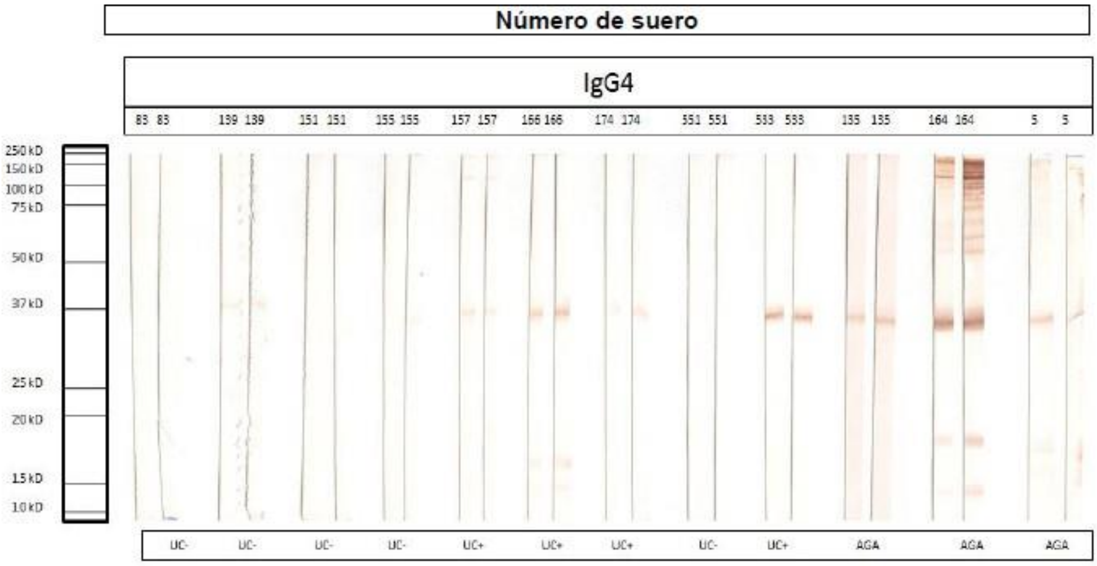

Figure S1B. Avidity of specific IgG4 in the sera studied (GAA, CU+, CU-, and Ctr). The nitrocellulose strip on the left was treated with urea.

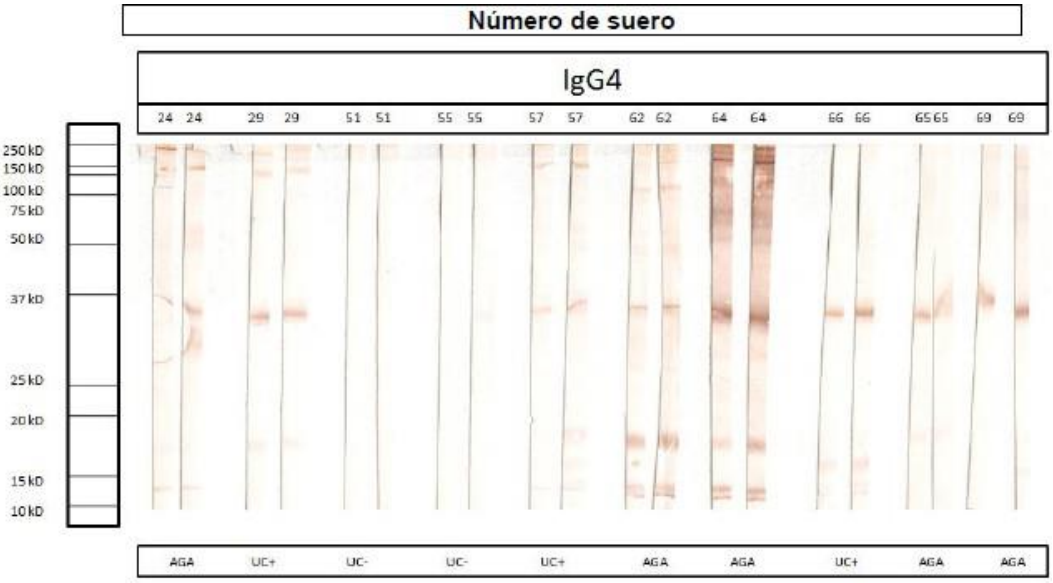

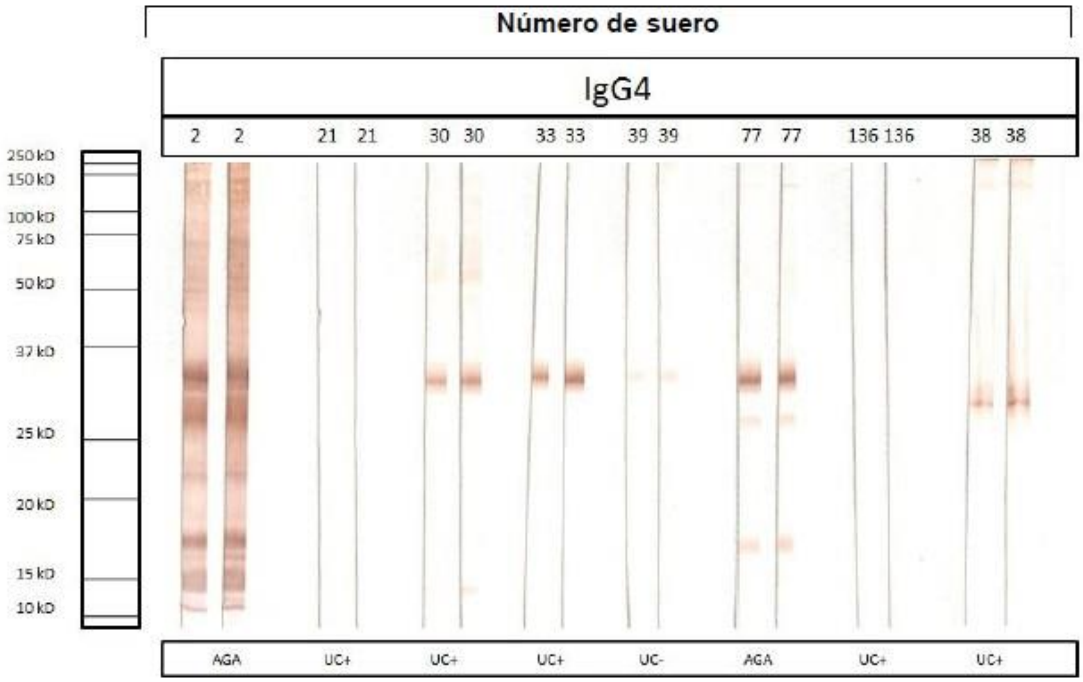

Figure S1C. Avidity of specific IgG4 in the sera studied (GAA, CU+, CU-, and Ctr). The nitrocellulose strip on the left was treated with urea.

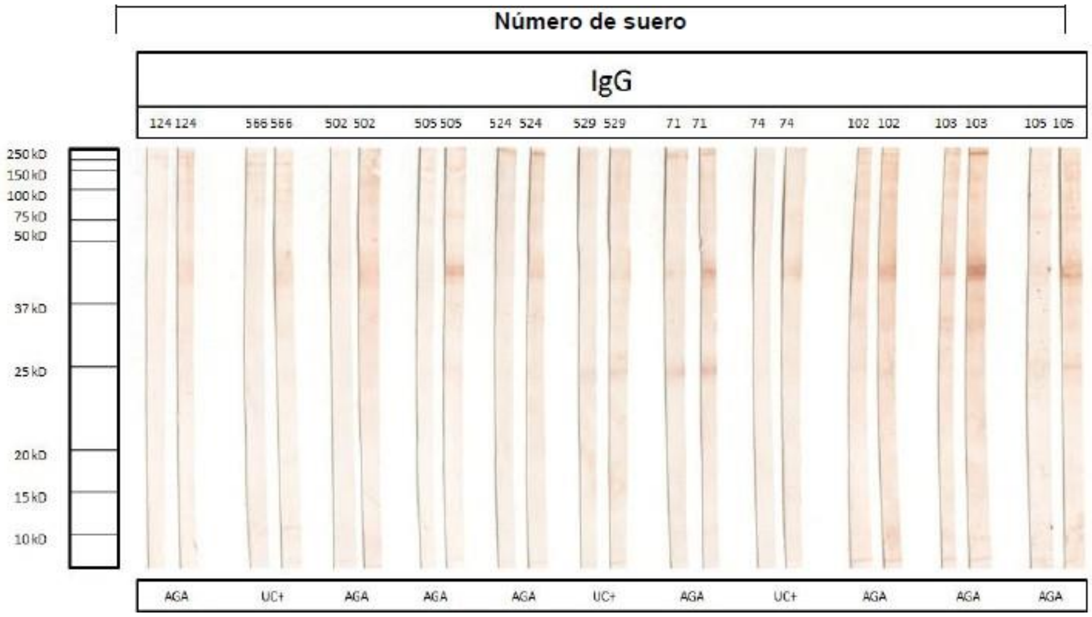

Figure S1D. Study of the avidity at the level of specific IgG in the sera studied (GAA, CU+, CU-, and Ctr) against the total larval antigen of A. simplex. The nitrocellulose strip on the left is treated with urea.

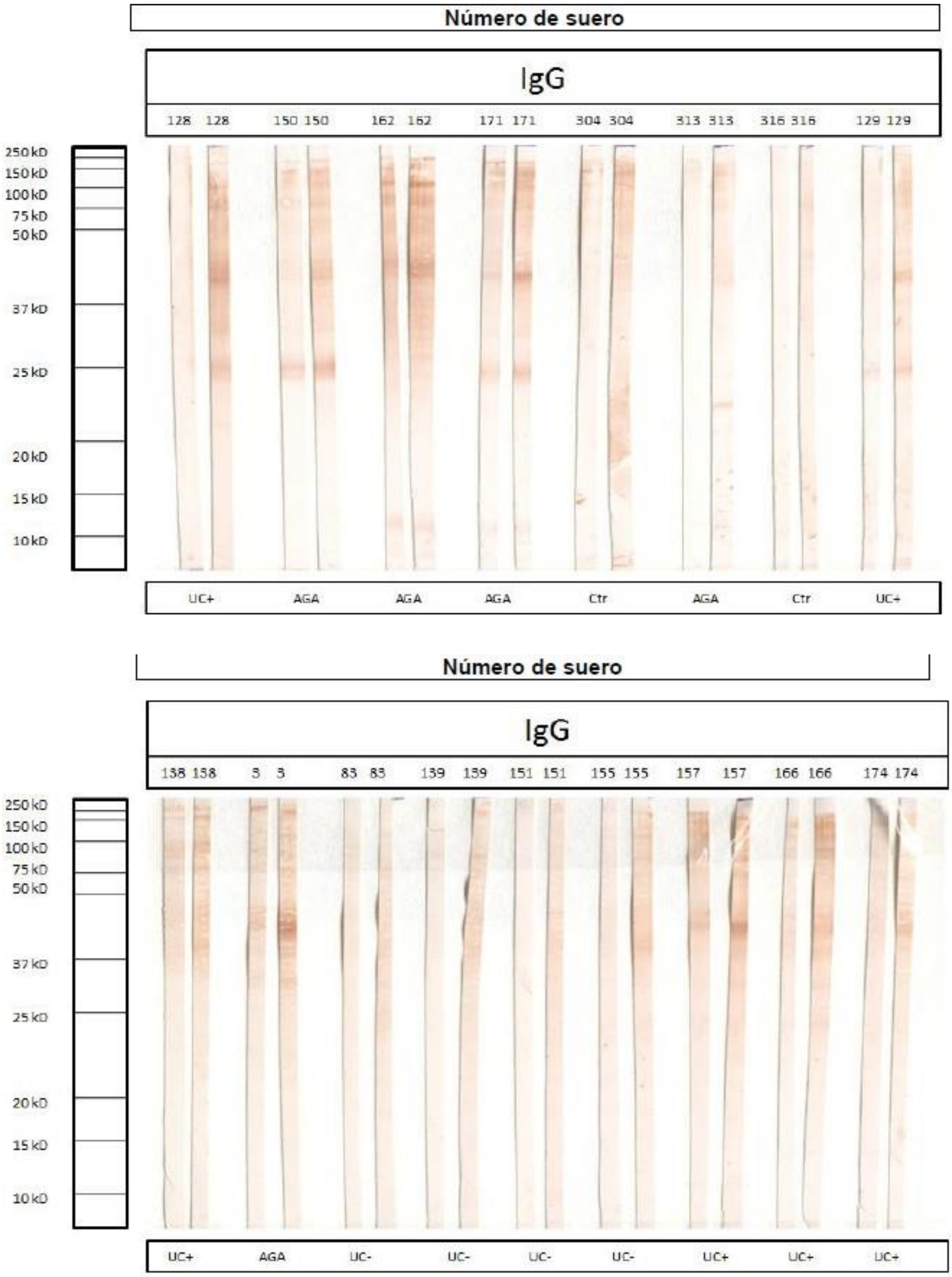

Figure S1E. Study of the avidity at the level of specific IgG in the sera studied (GAA, CU+, CU-, and Ctr) against the total larval antigen of A. simplex. The nitrocellulose strip on the left is treated with urea.

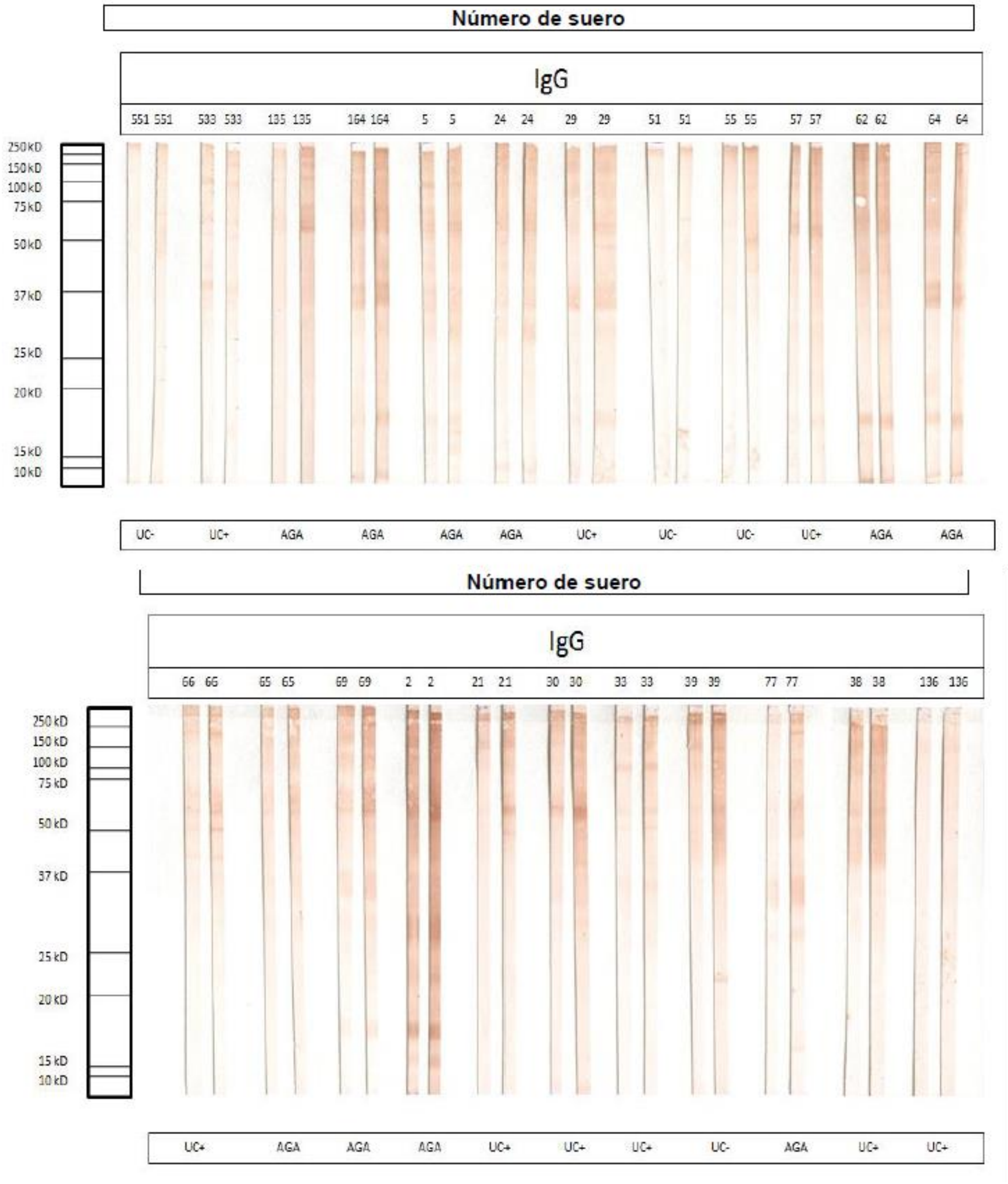

Figure S1F. Study of the avidity at the level of specific IgG in the sera studied (GAA, CU+, CU-, and Ctr) against the total larval antigen of A. simplex. The nitrocellulose strip on the left is treated with urea.

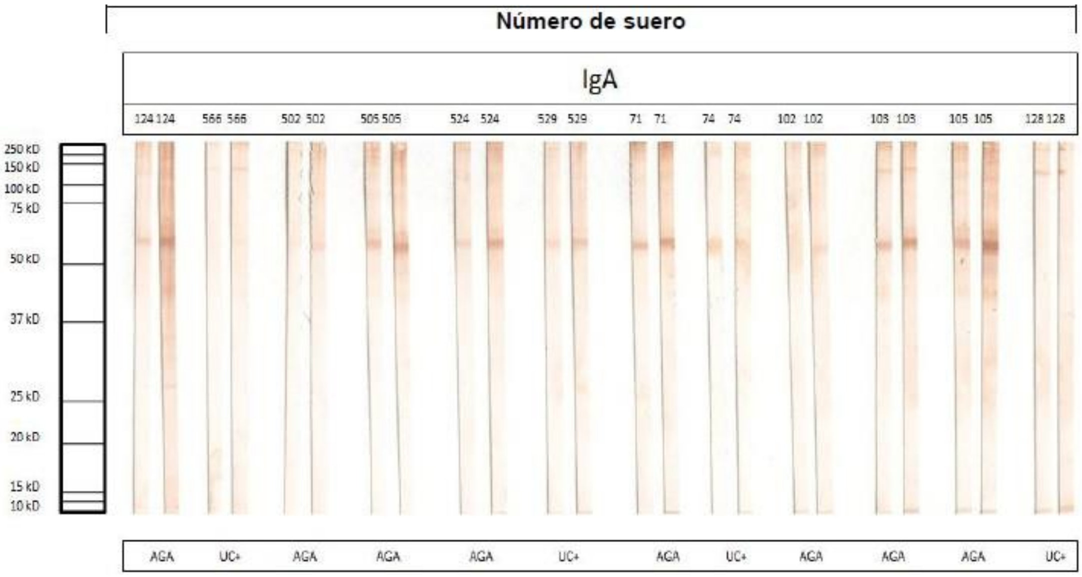

Figure S1G. Avidity index of specific IgA expressed as % avidity in the sera studied (GAA, CU+, CU-, and Ctr) against the total larval antigen of A. simplex. The nitrocellulose strips on the left are treated with urea.

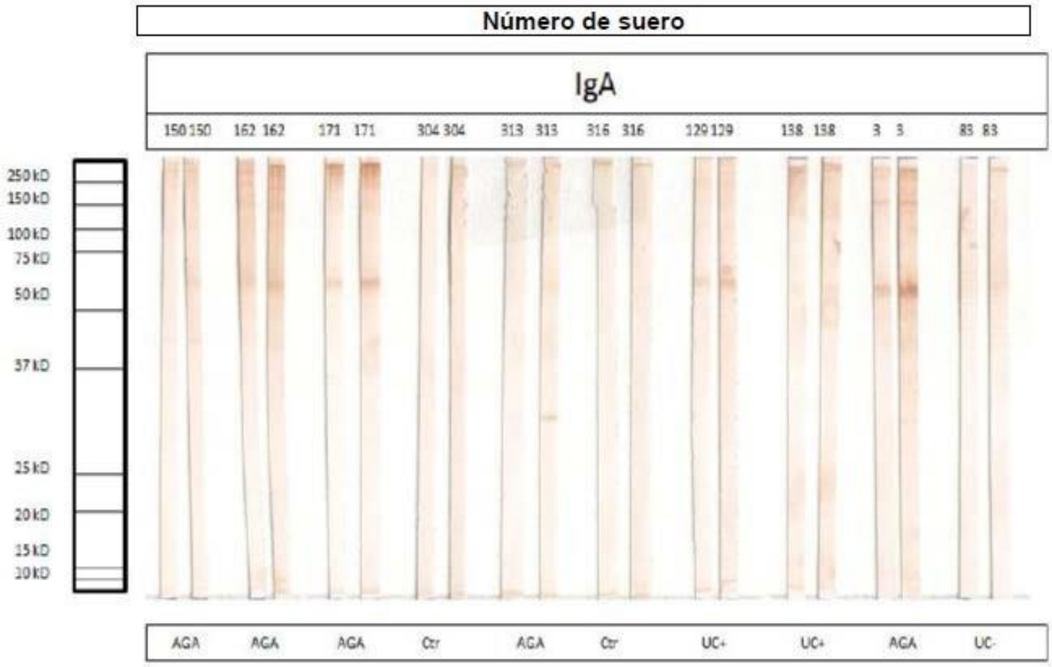

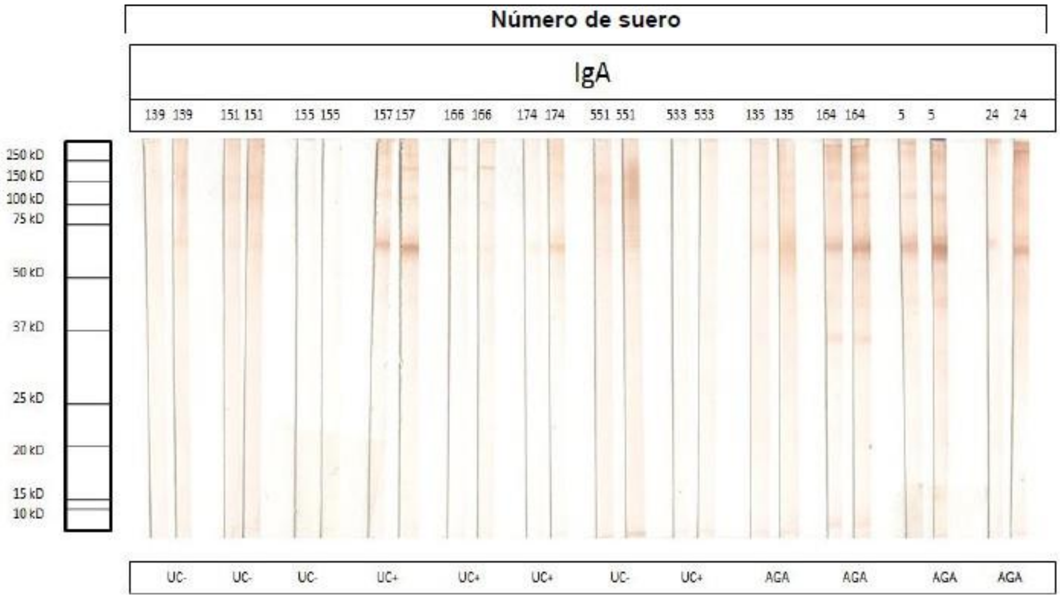

Figure S1H. Avidity index of specific IgA expressed as % avidity in the sera studied (GAA, CU+, CU-, and Ctr) against the total larval antigen of A. simplex. The nitrocellulose strips on the left are treated with urea.

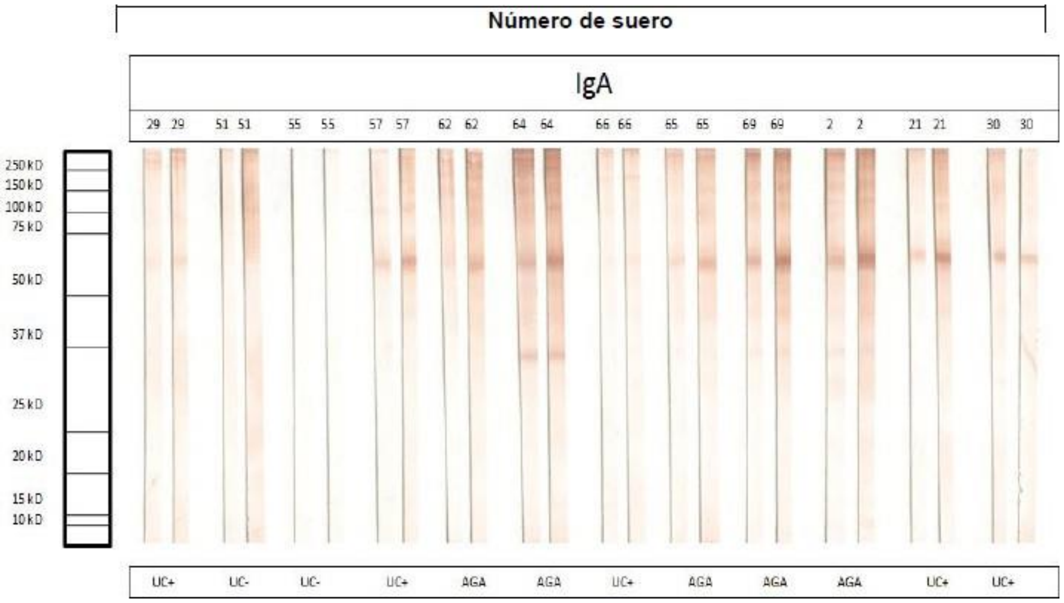

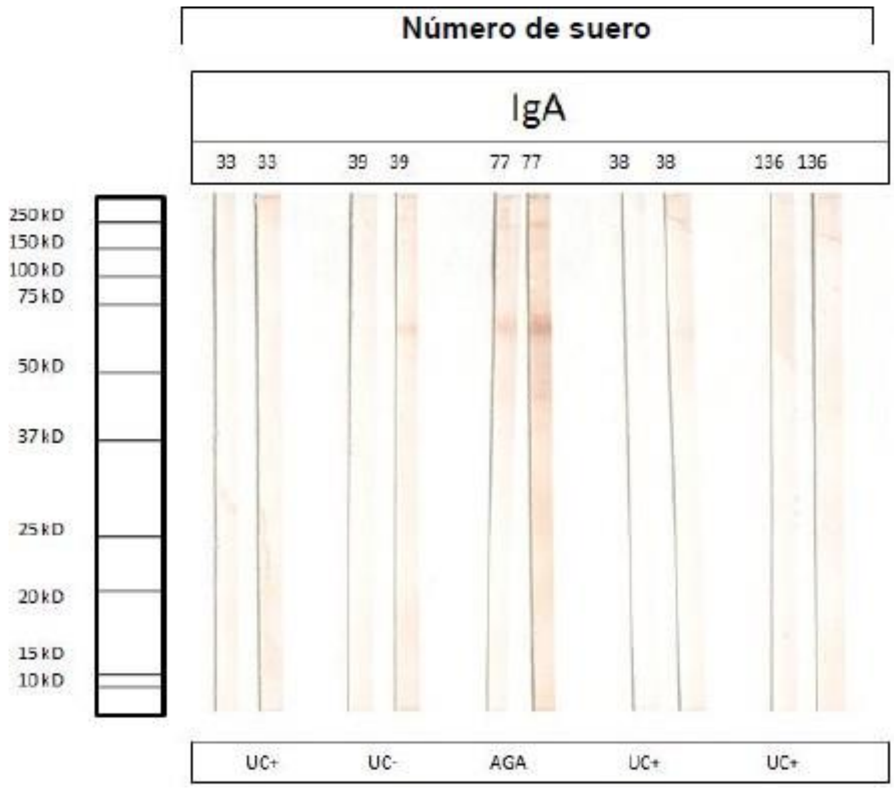

Figure S1I. Avidity index of specific IgA expressed as % avidity in the sera studied (GAA, CU+, CU-, and Ctr) against the total larval antigen of A. simplex. The nitrocellulose strips on the left are treated with urea.
